# A recessive coat color dilution in Dexter cattle attributed to a missense mutation in *SLC45A2*

**DOI:** 10.1111/age.70054

**Published:** 2025-10-16

**Authors:** Anna M. Fuller, Carol Davidson, Jessica L. Petersen

**Affiliations:** ^1^ Department of Animal Science University of Nebraska‐Lincoln Lincoln Nebraska USA; ^2^ Vancouver Island British Columbia Canada

**Keywords:** *Bos taurus*, melanogenesis, membrane‐associated transporter protein

## Abstract

Three colors of Dexter cattle are currently recognized: black, red, and dun. In Dexters, dun is determined by a recessive genotype of *TYRP1* (b/b) that dilutes an otherwise black animal (*MC1R* genotype ED/−); this variant does not impact red cattle. A subset of Dexters with dilute coat colors described as dark dun/chocolate (CD) or light dun/cream (CL) were identified. Although phenotypically similar to dun, they did not have the expected *TYRP1* b/b genotype. Given relationships among the reported individuals, we hypothesized that a novel recessive genotype is causative of CD on a black background and CL on a red background. Whole‐genome sequence was generated from four dilute Dexters (three CD and one CL), and one black calf of a CD dam. None of the cattle sequenced had the *TYRP1* b/b genotype. The comparison of variants in the five Dexter cattle to those from 226 non‐Dexter control cattle resulted in the identification of a missense variant in *SLC45A2* (NC_037347.1: g.39790189A>C; XM_002696386.6: c.398A>C) that fit the proposed hypothesis. Sanger sequencing of additional Dexter cattle (*n* = 227) demonstrated complete segregation of the recessive genotype with the CD and CL phenotypes. The mutation, predicted to result in a substitution of glutamine with proline (XP_002696432.2: p.Gln133Pro) in a transmembrane helix was classified as deleterious by SIFT. Further supporting its implication, *SLC45A2* is responsible for coat color dilutions and oculocutaneous albinism type IV in multiple species. Testing for the *SLC45A2* variant can be a valuable resource for Dexter breeders interested in coat color.

## INTRODUCTION

Dexter cattle originated in southern Ireland in the 1800s with over 200 imported into North America in the early 1900s (Plumb, 1920). The Dexter breed was initially considered an offshoot of the Kerry and Devon breeds, although genetic evidence suggests that only historically bred Dexter cattle show considerable influence from the Kerry breed. Both breeds are descendants of the Celtic black cattle (Bray et al., 2009). Given their relatively small size (mature height often under 1.2 m), hardiness, and dual‐purpose use, Dexter cattle are now found across the world. In the American Dexter Cattle Association and other Dexter cattle registries, recognized coat colors of the breed include black, red, and dun. Dexter cattle breeders who are interested in coat color can genotype their cattle to classify variation in two genes, melanocortin 1 receptor (*MC1R*) and tyrosinase related protein 1 (*TYRP1*), to identify what color variants they might carry.

**FIGURE 1:**
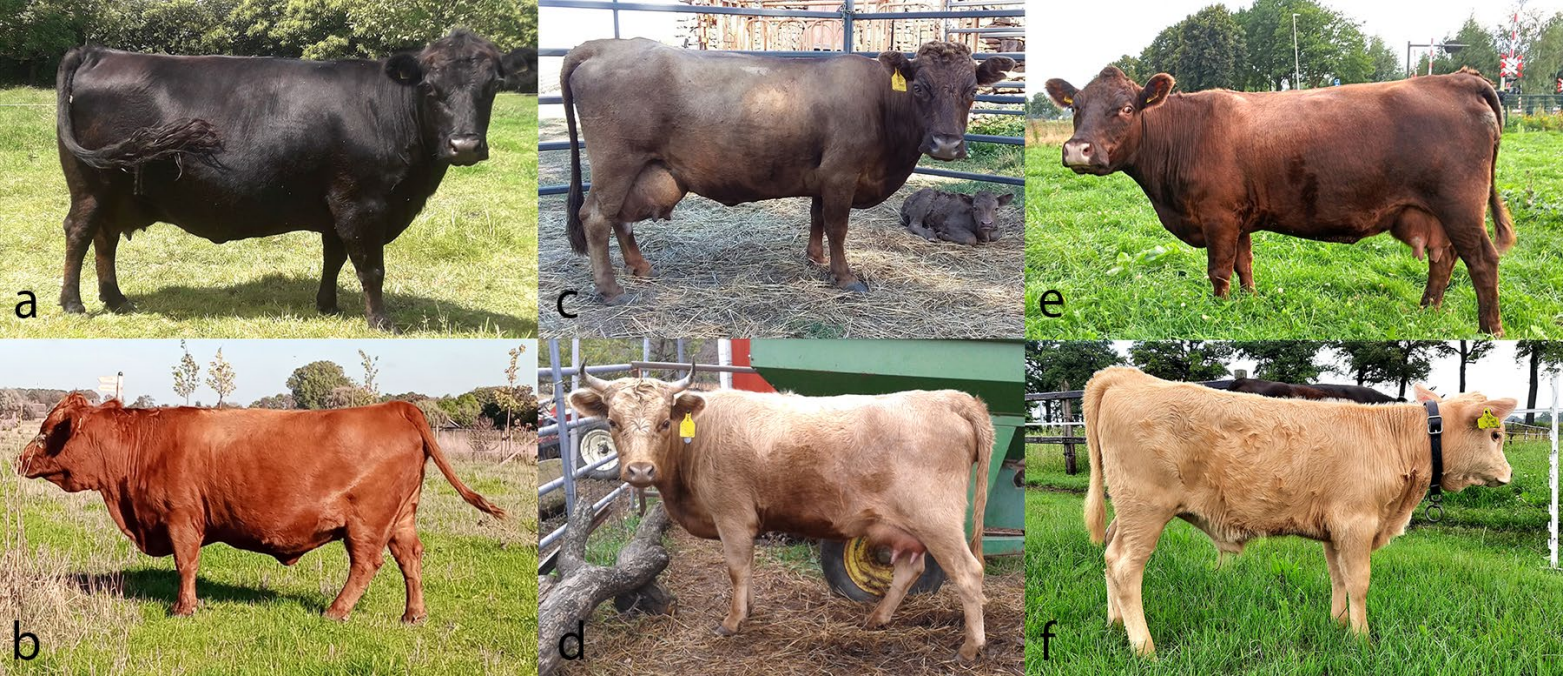
Photographs of Dexter cattle exhibiting (a) black, (b) red, (c, d) *TYRP1*‐dun, (e) chocolate (cd), or (f) cream (cl) coat colors.

In cattle, the *MC1R* extension locus is a primary determinant of coat color. A single point mutation from the reference (E+) results in dominant black (ED/−) and explains the black coat color in Dexter cattle (Figure 1a). Two copies of a single base pair, frameshift deletion results in recessive red (e) (Klungland et al., 1995). Several other variants in *MC1R* have been identified, including, ev1 and ev2, which also result in a recessive red phenotype (Hauser et al., 2022). Dexter cattle lacking the ED allele at *MC1R* are reported as and appear red (Figure 1b). When two copies of the *TYRP1* Dexter‐specific gene variant (b/b) are present on a black background (*MC1R* ED/−), the coat is diluted to a dun phenotype (Figure 1c,d; Berryere et al., 2003). The *TYRP1* mutation has no effect on the otherwise red cattle. In the current study, Dexter cattle lacking the dun *TYRP1* recessive genotype were reported with an unexpected dark dun/chocolate (CD; Figure 1e) or light dun/cream (CL; Figure 1f) coat color. The goal of this study was to identify the genetic basis of these coat colors. Based on observed inheritance patterns, we hypothesized that a novel recessive genotype is causative of CD on a black background and CL on a wildtype or red background.

## MATERIALS AND METHODS

### Sample collection

In total, 227 Dexter cattle samples were received from Australia (*n* = 39), Canada (*n* = 4), Europe (*n* = 85), and the USA (*n* = 99). The samples were of three types: whole EDTA blood (*n* = 1), hair (*n* = 212), and semen (*n* = 14).

### DNA isolation

The Gentra Puregene Blood Core Kit B (Qiagen, Venlo, the Netherlands) with a modified protocol was used to isolate DNA from whole EDTA blood (Sieck et al., 2020) and semen (Petersen et al., 2019). The Quick‐DNA Miniprep or Microprep Plus Kit (Zymo Research, Irvine, CA, USA) using the manufacturer's protocol was used to isolate DNA from hair follicles. DNA was quantified using an Epoch 2 Microplate Reader (BioTek, Winooski, VT, USA).

### Whole‐genome sequencing

Isolated DNA from three CD Dexter cattle, the black calf of one of the CD dams, and one CL steer was sent to Admera Health (South Plainfield, NJ, USA) for KAPA Hyper Prep library preparation (Roche) and Illumina NovaSeq 150 bp, paired‐end whole‐genome sequencing (WGS) to a targeted depth of 15× per animal. Raw data were processed by trimming adapters and removing poor‐quality bases using TrimGalore (Krueger et al., 2023). Reads were mapped to the ARS‐UCD1.2 genome with BWA‐MEM (Li & Durbin, 2009), and duplicates were marked with SAMtools (Danecek et al., 2021). GATK realigned indels and variants were called using the GATK Haplotype Caller (Van der Auwera & OConnor, 2020). A combined variant call file (VCF) merged the data of the five Dexter cattle with 226 multibreed control cattle previously sequenced from other projects (see Data availability). Initially, data were filtered using SnpSift to identify loci where the 4 CD/CL cattle had an alternative allele, while also assuming the control cattle from other breeds would only have the reference allele. Any locus that deviated from expected inheritance from the CD dam to her black offspring was further investigated using Integrative Genomics Viewer (Robinson et al., 2011); incorrect genotypes were corrected or flagged as appropriate (i.e., low read depth resulting in a potential missed heterozygote). Loci that could not be resolved were removed from consideration. The VCF was further filtered for homozygosity of the alternate allele in CD/CL cattle and heterozygosity of the CD offspring as expected for a recessive phenotype. Predicted variant impact was evaluated using the Ensembl Variant Effect Predictor (VEP; McLaren et al., 2016). Assuming that the causative variant is unique to Dexter cattle and relatively rare within the breed, variants that were previously reported in the NCBI Reference Sequence Database (RefSeq) were considered unlikely to be causative and eliminated from consideration. Variants without RefSeq identifiers were queried in public WGS data from 243 cattle (Heaton et al., 2016), which included four Dexters. The presence and frequency of the variants was also queried in a database of over 5500 cattle maintained by the University of Missouri (Dr. Robert Schnabel, personal communication). The predicted function of the final candidate variant was evaluated with SIFT (Ng, 2003) and PolyPhen2 (Adzhubei et al., 2010). The NIH NCBI protein blast alignment tool was used to evaluate amino acid conservation across multiple species (Altschul et al., 1990). The position of the variant of interest within the protein was investigated using UniProt (The UniProt Consortium, 2025) and DeepTMHMM (Hallgren et al., 2022).

### Sanger sequencing

Primers for coat color variants in melanocortin 1 receptor (*MC1R*), tyrosinase related protein 1 (*TYRP1*), and solute carrier family 45 member 2 (*SLC45A2*) were designed in Primer3 (Untergasser et al., 2012) (Table S2). PCR was carried out in a reaction volume of 12 μL, containing 4 μL of 5 ng/μL DNA, 0.75 μL 20 μM forward primer, 0.75 μL 20 μM reverse primer, 4.45 μL MilliQ water, 0.25 μL 25 mM MgCl_2_, 1.2 μL 10× PCR Reaction Buffer with 20 mM MgCl_2_, 0.5 μL PCR Nucleotide Mix, and 0.1 μL 5 U/μL FastStart Taq DNA Polymerase from the Roche FastStart kit (Sigma‐Aldrich, St. Louis, MO, USA). PCR was performed with the following cycle conditions: 94°C for 4 min, 32 cycles of 94°C for 30 s, annealing temperature (Table S1) for 30 s, 72°C for 45 s, a final extension at 72°C for 10 min, and then held at 10°C. After checking PCR products on a 1.2% agarose gel, 3 μL PCR products were added to 0.75 μL ExoSAP‐IT (Applied Biosystems, Foster City, CA, USA) and 13.25 μL MilliQ water. Purification was performed with the following cycler conditions: 37°C for 30 min, 80°C for 15 min, and then held at 15°C. A 2‐μL aliquot of 20 μM primer was added before sending to Eurofins Genomics (Louisville, KY, USA) for Sanger sequencing on an ABI 3730 xl. Sequencing results were analyzed using Sequencher 5.4.6 (Gene Codes Corporation, Ann Arbor, MI, USA).

## RESULTS

### Whole‐genome sequencing

After WGS data were processed, an average of 13.5× coverage was achieved in the five samples (13.2–13.9×). Filtering to include only variants present in the CD/CL cattle and not present in control non‐Dexter cattle identified 170 initial candidate variants. Integrative Genome Viewer analysis removed eight loci that did not fit Mendelian expectations between the CD dam and black offspring. All 162 remaining variants were within a 1.8‐Mb segment of Chromosome 20 (NC_037347.1) with 161 fitting the hypothesized recessive mode of inheritance (Table S3); 142 were known variants (RefSeqGene; Goldfarb et al., 2025). When the remaining 19 variants without prior annotation (Table S4) were queried against the University of Missouri's WGS database, all but six were present in multiple other breeds; two were not present in any other cattle (Table 1). Of those two variants, only solute carrier family 45 member 2 (*SLC45A2*) had a predicted impact on gene function.

**TABLE 1:** Candidate variants identified with no prior observation in other cattle.

Chr	Position (bp)	Reference	Alternative	Variant annotation	Gene ID
20	38 156 214	TC	T	Intronic	*UGT3A2*
20	39 790 189	A	C	Missense variant	*SLC45A2*

Sanger sequencing to genotype the *SLC45A2* variant as well as known variation in *MC1R* (ED, E+, e, ev1, ev2) and *TYRP1* (Figure S1) was performed on an additional 222 Dexter DNA samples (Table 2; Table S5). Genotyping confirmed that all Dexter cattle phenotypically described as dun or chocolate were homozygous for either the known *TYRP1* variant (b/b), or for the newly identified *SLC45A2* variant (NC_037347.1: g.39790189A>C) with the presence of at least one ED allele at *MC1R* (base color = black). The six Dexter cattle identified as cream had an E+/E+, E+/e, or e/e genotype at *MC1R* and were homozygous for the *SLC45A2* variant.

**TABLE 2:** Sanger sequencing and whole‐genome sequencing results of the candidate *SLC45A2* variant and known Dexter coat color variants in *MC1R* and *TYRP1*.

	*MC1R*					*TYRP1*			*SLC45A2*		
	ED/−	E+/−	e/e	e/ev1	ev1/ev1	B/B	B/b	b/b	A/A	A/C	C/C
Chocolate	8	–	–	–	–	7	1	–	–	–	8
Cream	–	5	1	–	–	6	–	–	–	–	6
	(cases, *n* = 14)										
Black	100	–	–	–	–	41	59	–	76	24	–
Red	–	34	16	2	6	26	17	15	40	18	–
Dun	55	–	–	–	–	–	–	55	52	3	–
	(controls, *n* = 213)										

### Predicted impact on protein function

The cattle *SLC45A2* gene spans 2.3 kb on chromosome 20 of ARS‐UCD1.2 (NC_037347.1). The missense variant found in exon 1 of *SLC45A2* (XM_002696386.6: c.398A>C) was predicted to result in the substitution of glutamine with proline (XP_002696432.2: p.Gln133Pro) in what is annotated by UniProt as a helical transmembrane domain (The UniProt Consortium, 2025). This annotation is supported by DeepTMHMM, which further specifies that the reside resides in the second of 12 transmembrane domains (Hallgren et al., 2022). The mutation is classified as deleterious by SIFT with a score of 0.01 and as probably damaging with a score of 0.999 by PolyPhen‐2. The alignment of the SLC45A2 amino acid sequence indicated that the site of the variant is conserved across all species included in the analysis (Figure S1).

## DISCUSSION

A novel recessive variant in *SLC45A2* was identified in Dexter cattle with a dilute coat color, unrelated to the previously identified *TYRP1* dun dilution. The phenotypic resemblance of CD and CL cattle to those with the *TYRP1*‐based dun dilution probably contributed to its previously undetected presence in the population. The *SLC45A2* variant segregated perfectly in all the studied Dexter cattle that were classified as being dilute in color, but which did not have the *TYRP1* b/b genotype. The recessive homozygous genotype of *SLC45A2* was not identified in any non‐dilute cattle. As additional support for its association with color in these cattle, the SLC45A2 protein, also known as membrane‐associated transporter protein or antigen isolated from immunoselected melanoma 1, is involved in regulating coat, eye, and skin color in at least 18 other species (OMIA: *SLC45A2* (606202); https://doi.org/10.25910/2AMR‐PV70). The effect of each variant on coat or hair color, however, varies. For example, mutations in *SLC45A2* in humans can result in hair color ranging from white to brown (Inagaki et al., 2004). In horses, a missense mutation in exon 2 of *SLC45A2* results in partial dilution of red pigment to a cream phenotype in heterozygotes (“cream” dilution), while homozygosity leads to the dilution of black pigment and further dilutes red pigment (Mariat et al., 2003). The color dilution associated with the pearl coat color in horses requires homozygosity of the causative variant in *SLC45A2*, consistent with the mechanism observed for the Dexter *SLC45A2* variant (Holl et al., 2019). An unexpected outcome of this study, allele ev1 of *MC1R*, was identified in our sample of Dexter cattle. To our knowledge, this variant had not been previously described in the breed although the publication in which it was first described identified its presence in 16 European breeds, although often at low frequency (Hauser et al., 2022).

During development, melanoblasts migrate into various regions of the body, with some developing into melanocytes found in hair follicles (Bonaventure et al., 2013). A mix of melanin, including eumelanin (black‐brown) or pheomelanin (red‐yellow) (Ito & Wakamatsu, 2011), is produced by melanosomes within melanocytes and contributes pigment to growing hair shafts (Botchkareva et al., 2003). In black cattle, alpha‐melanocyte‐stimulating hormone is bound to the G‐protein coupled receptor, MC1R, initiating a downstream cAMP‐dependent signaling pathway to regulate microphthalmia‐associated transcription factor (MITF) production within the melanocyte (D'Mello et al., 2016). MITF then binds genes associated with pigmentation, such as tyrosinase (*TYR*) (Yasumoto et al., 1994), DOPAchrome tautomerase (*DCT*), and *TYRP1* (Bertolotto et al., 1998). MITF acts through an indirect regulatory mechanism, on *SLC45A2* (Du & Fisher, 2002).

**FIGURE 2:**
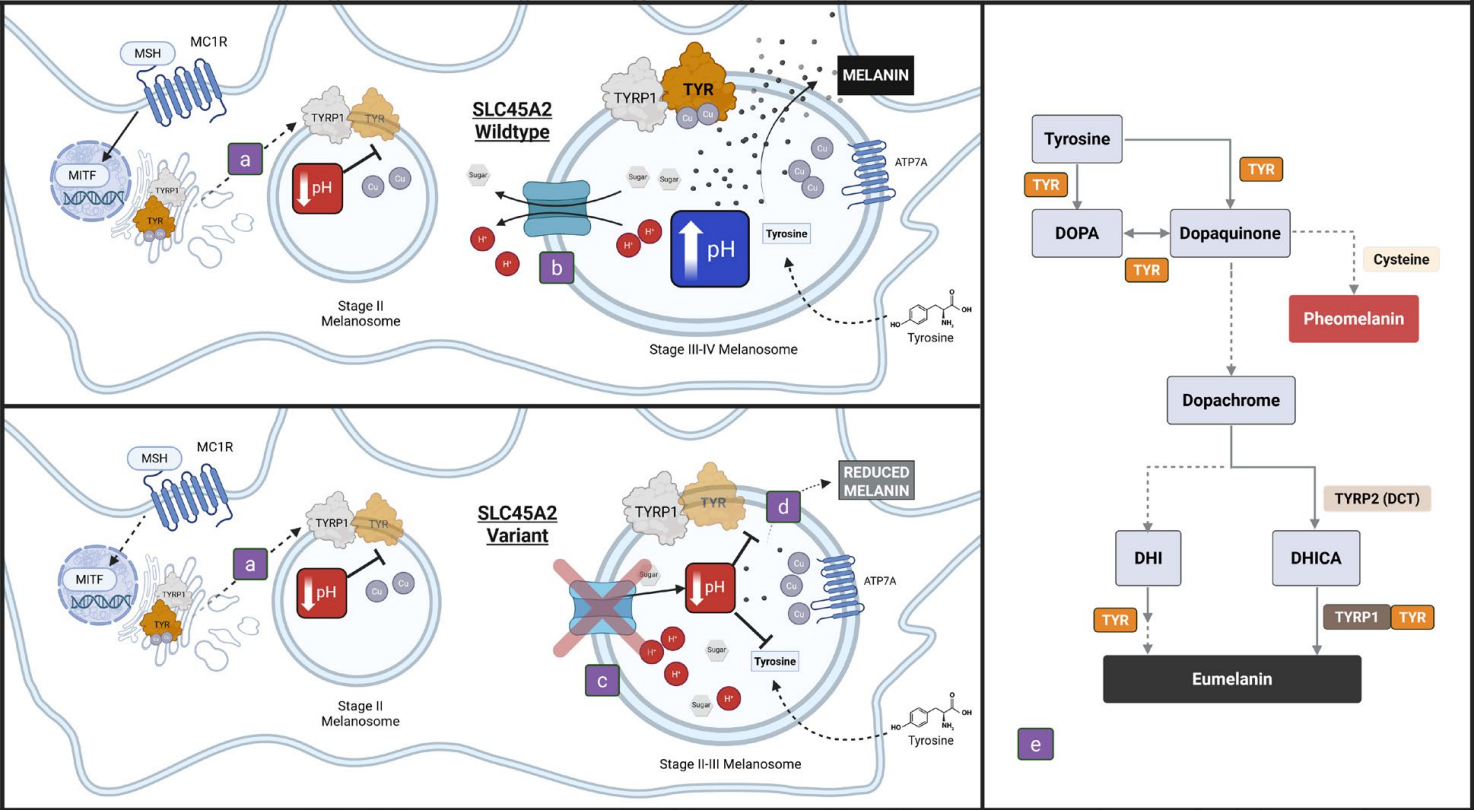
The hypothesized role of the Dexter *SLC45A2* variant in the melanogenic pathway. Early melanogenesis involves activation of *MITF* and thus the expression of *TYRP1* and *TYR*. (a) TYRP1 and TYR are trafficked to an acidic stage II melanosome. As the melanosome matures, the wildtype SLC45A2 cotransporter (b) exports protons, increasing the internal pH of the melanosome. This pH shift allows copper to bind tyrosinase, activating the enzyme to catalyze the initial steps of melanin synthesis. The Dexter *SLC45A2* variant (c) fails to regulate pH, and subsequently reduces tyrosinase activity (d), ultimately preventing the enzymatic conversion of tyrosine into melanin intermediates (e). Created in BioRender. Fuller, A. (2025) https://BioRender.com/yjfzjrs.

TYR and TYRP1 are trafficked to the membrane of immature melanosomes from the trans‐Golgi network (Figure 2a) (Watabe et al., 2004). Within the melanosome, a complex biochemical pathway converts tyrosine to melanin, beginning with hydroxylation of tyrosine to 3,4‐dihydroxyphenylalanine by TYR (Figure 2e) (Slominski et al., 2005). TYR enzymatic activity requires pH mediated binding of copper ions (Bin et al., 2015) and is optimal at near neutral pH (Ancans et al., 2001). SLC45A2 is a secondary active transporter belonging to the proton/glucose cotransporter family that localizes to mature melanosomes within subdomains of the outer membrane (Bin et al., 2015, 2016; Le et al., 2020). The SLC45A2 transporter expels protons and sugar molecules from the melanosomal lumen into the cytosol during stage III‐IV melanogenesis, actively increasing pH (Figure 2b) (Le et al., 2020; Liu et al., 2022). We hypothesize that the variant identified in this study impacts SLC45A2 transport function (Figure 2c), resulting in a more acidic pH and reducing tyrosinase activity, leading to a reduction in melanin synthesis (Figure 2d).

Like SLC45A2, TYRP1 modulates TYR activity, but through a different mechanism. Together with DCT, TYRP1 stabilizes TYR catalytic function (Kobayashi et al., 1998), positively regulates TYR efficient targeting to melanosomes (Nakamura & Fukuda, 2024), and is important in the 5,6‐dihydroxyindole‐2‐carboxylic acid‐melanin production pathway (Figure 2e) (Sarangarajan & Boissy, 2001). The phenotypic impact of the Dexter‐specific *TYRP1* variant on only eumelanic cattle (Berryere et al., 2003) may be due to compensatory pathways, redundant genes, or its specific role in 5,6‐dihydroxyindole‐2‐carboxylic acid‐melanin synthesis. The knockdown of *SLC45A2* in human melanocytes showed melanosomes stalled at stage II (Bin et al., 2015), which suggests that SLC45A2 has an earlier impact on both eumelanin and pheomelanin synthesis than TYRP1 and may explain its impact on both black and red cattle.

In this study, no cattle were homozygous for both the *SLC45A2* and *TYRP1* dilution genotypes. Given the different functions of the two genes, it is of interest whether the presence of both variants results in a more extreme coat color dilution on otherwise black cattle. Further, developmental ocular abnormalities (oculocutaneous albinism type 4) result from *SLC45A2* mutations including cases in dogs (Caduff et al., 2017; Wijesena & Schmutz, 2015; Winkler et al., 2014), a gorilla (Prado‐Martinez et al., 2013), and numerous instances in humans (OMIM: 606574, https://omim.org). In Braunvieh cattle, two mutations in *SLC45A2* were identified in two half‐sibling calves with oculocutaneous albinisms (Rothammer et al., 2017). Dexter cattle with CD/CL or *TYRP1*‐dun phenotypes have been described by owners to exhibit dilute (hazel or gold) eye pigments although to our knowledge no issues with their sight have been reported. This observation poses additional questions for future study on how these variants may alter ocular characteristics in dilute Dexter cattle.

## AUTHOR CONTRIBUTIONS

**Anna M. Fuller:** Investigation; writing – original draft; methodology; validation; visualization; formal analysis; data curation. **Carol Davidson:** Conceptualization; resources. **Jessica L. Petersen:** Investigation; funding acquisition; writing – review and editing; methodology; project administration; supervision.

## FUNDING INFORMATION

This project was funded by Wakarusa Ridge Ranch, the American Dexter Cattle Association, and the Carol Davidson estate.

## CONFLICT OF INTEREST STATEMENT

Testing for this variant is available through the UC Davis Veterinary Genetics Laboratory, which may, for a limited time, provide a portion of testing proceeds to the Petersen Lab as in‐kind research support or other consideration.

## ETHICS STATEMENT

All procedures adhered to the guidelines of the University of Nebraska‐Lincoln Institutional Animal Care and Use Committee. No committee approval was necessary as all samples were collected and contributed by the animals' owners.

## Supporting information


Table S1.

## Data Availability

WGS files are available at the NCBI Sequence Read Archive (accession PRJNA1285965). Control data are found in accessions PRJNA1042650, PRJNA1042814, PRJNA513064, PRJNA663547, PRJNA1013498, PRJNA994471, and PRJNA1026143.
