# Supplementary material for: A recessive coat color dilution in Dexter cattle attributed to a missense mutation in SLC45A2
Source: Anim Genet. 2025 Oct 16;56(5):e70054. doi: 10.1111/age.70054 (PMC12530945; doi:10.1111/age.70054)
Supplement: Supplementary file 1 — Table S1. [file AGE-56-0-s001.docx]

**Supplement: A recessive coat color dilution in Dexter Cattle attributed to a missense mutation in *SLC45A2***

Supplementary Table S1. Primer sequences and annealing temperatures.

| Gene | Primer | Sequence | Annealing Temperature (°C) |
| --- | --- | --- | --- |
| *MC1R* | Forward  Reverse | 5'- CAT GAG TTG AGC AGG ACC CT -3'  5'- AGC ATG TGG ACG TAG AGG AC -3' | 60 |
| *SLC45A2* | Forward  Reverse | 5'- AGT CTC CCC TAC GCA CCT AT -3'  5'- GCA CAC ACA CAA AAC CCC AT -3' | 58 |
| *TYRP1* | Forward  Reverse | 5'- CCA GCT CAA CCA GGT CAG AA -3'  5'- TCA TGC AAT TCC CAG TTC TGC -3' | 56 |

Supplementary Table S2. Variant information for genes of interest (ARS-UCD1.2).

| Gene | Variant | Chr | Position | Reference | Alternative | Variant Annotation |
| --- | --- | --- | --- | --- | --- | --- |
| *TYRP1* | b | 8 (NC_037335.1) | 31633328 | G | A | Missense variant |
| *MC1R* | e | 18 (NC_037345.1) | 14705685 | G | del | Frameshift deletion |
| *MC1R* | ED | 18 (NC_037345.1) | 14705671 | T | C | Missense variant |
| *MC1R* | ev1 | 18 (NC_037345.1) | 14705799 | C | T | Missense variant |
| *MC1R* | ev2 | 18 (NC_037345.1) | 14705638 | G | A | Missense variant |

Supplementary Table S3. Candidate variants that fit the hypothesized mode of homozygous recessive inheritance identified with whole-genome sequencing data. All variants are on ARS-UCD1.2 chromosome 20 (NC_037347.1).

| Chr | Position (bp) | Reference | Alternative | Variant Annotation | Gene ID | RefSeq ID |
| --- | --- | --- | --- | --- | --- | --- |
| 20 | 37990483 | A | T | Intronic | *RANBP3L* | rs470123037 |
| 20 | 37995958 | T | C | Downstream gene variant | *RANBP3L* | - |
| 20 | 38006184 | C | T | Intronic | *NADK2* | - |
| 20 | 38040367 | G | T | Intronic | *SKP2* | - |
| 20 | 38052636 | G | A | Intronic | *SKP2* | - |
| 20 | 38156214 | TC | T | Intronic | *UGT3A2* | - |
| 20 | 38303425 | T | A | Intronic | *IL7R* | rs383349355 |
| 20 | 38332310 | A | G | Intronic | *IL7R* | rs378244618 |
| 20 | 38344182 | C | T | Intergenic | *-* | rs211642698 |
| 20 | 38379046 | C | G | Intronic | *SPEF2* | rs381801006 |
| 20 | 38390770 | C | T | Intronic | *SPEF2* | rs383763257 |
| 20 | 38526995 | T | C | Intronic | *SPEF2* | rs382057310 |
| 20 | 38541369 | A | G | Intronic | *SPEF2* | - |
| 20 | 38638882 | T | G | Intergenic | - | - |
| 20 | 38655483 | T | C | Intergenic | - | - |
| 20 | 38659643 | T | C | Intergenic | - | rs383426494 |
| 20 | 38727245 | C | A | Intergenic | - | rs380130820 |
| 20 | 38757375 | A | G | Intergenic | - | rs378017779 |
| 20 | 38786347 | C | T | Intergenic | - | rs448321114 |
| 20 | 38792583 | A | C | Intergenic | - | rs381843478 |
| 20 | 38793995 | C | T | Intergenic | - | rs435693461 |
| 20 | 38794056 | G | A | Intergenic | - | rs455689837 |
| 20 | 38794192 | C | T | Intergenic | - | rs384357596 |
| 20 | 38794833 | A | AT | Intergenic | - | rs380567926 |
| 20 | 38794836 | A | AT | Intergenic | - | rs800437394 |
| 20 | 38794869 | A | T | Intergenic | - | rs383551713 |
| 20 | 38795583 | T | C | Intergenic | - | rs382036586 |
| 20 | 38795587 | T | C | Intergenic | - | rs380284227 |
| 20 | 38795905 | T | TATATCC | Intergenic | - | rs460737690 |
| 20 | 38796198 | G | C | Intergenic | - | rs483135935 |
| 20 | 38796867 | C | T | Intergenic | - | rs380980687 |
| 20 | 38797362 | ACT | A | Intergenic | - | rs383206907 |
| 20 | 38797664 | A | G | Intergenic | - | rs208372429 |
| 20 | 38797718 | A | G | Intergenic | - | rs210410897 |
| 20 | 38798343 | T | A | Intergenic | - | rs381943724 |
| 20 | 38800241 | A | G | Intergenic | - | rs434504491 |
| 20 | 38800820 | C | T | Intergenic | - | rs382333251 |
| 20 | 38801024 | G | A | Intergenic | - | rs381121431 |
| 20 | 38801083 | T | C | Intergenic | - | rs716383913 |
| 20 | 38802493 | C | T | Intergenic | - | rs450487753 |
| 20 | 38802566 | A | G | Intergenic | - | rs384199088 |
| 20 | 38802847 | A | C | Intergenic | - | rs719513943 |
| 20 | 38803975 | G | T | Intergenic | - | rs470912185 |
| 20 | 38804373 | CT | C | Intergenic | - | - |
| 20 | 38804596 | G | A | Intergenic | - | rs460129631 |
| 20 | 38804752 | G | GA | Intergenic | - | - |
| 20 | 38805583 | G | A | Intergenic | - | rs464460991 |
| 20 | 38806673 | T | C | Intergenic | - | rs379437273 |
| 20 | 38806816 | G | C | Intergenic | - | rs383041585 |
| 20 | 38806879 | C | T | Intergenic | - | rs717222321 |
| 20 | 38806880 | A | G | Intergenic | - | rs433230506 |
| 20 | 38806900 | T | C | Intergenic | - | rs446940438 |
| 20 | 38807060 | G | A | Intergenic | - | rs466984004 |
| 20 | 38807224 | C | T | Intergenic | - | rs720848575 |
| 20 | 38807297 | C | A | Intergenic | - | rs1117574713 |
| 20 | 38807327 | G | C | Intergenic | - | rs1114414998 |
| 20 | 38807353 | C | T | Intergenic | - | - |
| 20 | 38807365 | A | G | Intergenic | - | - |
| 20 | 38807403 | AAG | A | Intergenic | - | - |
| 20 | 38808463 | C | A | Intergenic | - | rs384041208 |
| 20 | 38808793 | G | A | Intergenic | - | rs482304392 |
| 20 | 38809123 | G | A | Intergenic | - | rs384347501 |
| 20 | 38809145 | A | G | Intergenic | - | rs716336286 |
| 20 | 38809189 | G | A | Intergenic | - | rs385749849 |
| 20 | 38809358 | C | G | Intergenic | - | rs378538709 |
| 20 | 38809497 | G | A | Intergenic | - | rs378592375 |
| 20 | 38809571 | GT | G | Intergenic | - | rs801363658 |
| 20 | 38809728 | C | T | Intergenic | - | rs382750379 |
| 20 | 38809736 | T | C | Intergenic | - | rs379988916 |
| 20 | 38809787 | T | G | Intergenic | - | rs724040572 |
| 20 | 38809790 | T | A | Intergenic | - | rs458228382 |
| 20 | 38809986 | T | C | Intergenic | - | rs385555656 |
| 20 | 38810184 | A | C | Intergenic | - | rs385604063 |
| 20 | 38811117 | T | C | Intergenic | - | rs385042313 |
| 20 | 38811244 | C | T | Intergenic | - | rs472045245 |
| 20 | 38811268 | A | G | Intergenic | - | rs440717239 |
| 20 | 38811435 | T | C | Intergenic | - | rs383193799 |
| 20 | 38811662 | A | G | Intergenic | - | rs380541687 |
| 20 | 38811776 | G | A | Intergenic | - | rs383835676 |
| 20 | 38811866 | T | C | Intergenic | - | rs467536772 |
| 20 | 38812041 | G | A | Intergenic | - | rs383489626 |
| 20 | 38812123 | A | G | Intergenic | - | rs451989111 |
| 20 | 38812150 | G | A | Intergenic | - | rs434275601 |
| 20 | 38812170 | C | G | Intergenic | - | rs454362909 |
| 20 | 38812287 | A | G | Intergenic | - | rs381730337 |
| 20 | 38812645 | T | A | Intergenic | - | rs380059653 |
| 20 | 38812723 | T | C | Intergenic | - | rs438961243 |
| 20 | 38812754 | T | C | Intergenic | - | rs211331660 |
| 20 | 38812959 | A | AAC | Intergenic | - | rs475480804 |
| 20 | 38813076 | A | T | Intergenic | - | rs208557742 |
| 20 | 38813458 | C | A | Intergenic | - | rs382327428 |
| 20 | 38814016 | G | A | Intergenic | - | rs209558129 |
| 20 | 38814210 | G | A | Intergenic | - | rs380631127 |
| 20 | 38814279 | C | G | Intergenic | - | rs479579080 |
| 20 | 38814287 | G | A | Intergenic | - | rs721343965 |
| 20 | 38814288 | C | G | Intergenic | - | rs448045118 |
| 20 | 38814315 | T | C | Intergenic | - | rs211183308 |
| 20 | 38814797 | C | T | Intergenic | - | rs379349017 |
| 20 | 38814863 | A | G | Intergenic | - | rs474850650 |
| 20 | 38814951 | G | A | Intergenic | - | rs381845057 |
| 20 | 38815028 | T | C | Intergenic | - | rs380557176 |
| 20 | 38815035 | G | A | Intergenic | - | rs378532521 |
| 20 | 38815469 | A | G | Intergenic | - | rs481772225 |
| 20 | 38815916 | A | G | Intergenic | - | rs437125141 |
| 20 | 38815962 | G | A | Intergenic | - | rs457196075 |
| 20 | 38816426 | T | C | Intergenic | - | rs473233656 |
| 20 | 38816647 | A | G | Intergenic | - | rs441846458 |
| 20 | 38816657 | C | T | Intergenic | - | rs461795333 |
| 20 | 38817011 | T | A | Intergenic | - | rs384378895 |
| 20 | 38817046 | T | G | Intergenic | - | rs385128181 |
| 20 | 38817629 | C | T | Intergenic | - | rs797138285 |
| 20 | 38817911 | G | A | Intergenic | - | rs385612285 |
| 20 | 38818832 | G | A | Intergenic | - | rs380543840 |
| 20 | 38818896 | C | T | Intergenic | - | rs442319827 |
| 20 | 38819535 | T | G | Intergenic | - | rs437757026 |
| 20 | 38824550 | G | GA | Intergenic | - | rs481704585 |
| 20 | 38825100 | T | C | Intergenic | - | rs437191039 |
| 20 | 38825644 | A | C | Intergenic | - | rs481831467 |
| 20 | 38825956 | G | A | Intergenic | - | rs385866409 |
| 20 | 38826369 | C | T | Intergenic | - | rs433104491 |
| 20 | 38827991 | G | A | Intergenic | - | rs382022004 |
| 20 | 38828031 | C | T | Intergenic | - | rs478161765 |
| 20 | 38828630 | T | G | Intergenic | - | rs382969632 |
| 20 | 38828867 | G | C | Intergenic | - | rs207482820 |
| 20 | 38829035 | C | G | Intergenic | - | rs383770109 |
| 20 | 38829231 | A | G | Intergenic | - | rs207823358 |
| 20 | 38829677 | G | A | Intergenic | - | rs207905254 |
| 20 | 38829712 | G | T | Intergenic | - | rs384192132 |
| 20 | 38829956 | C | T | Intergenic | - | rs211393482 |
| 20 | 38830071 | T | C | Intergenic | - | rs208060620 |
| 20 | 38830352 | C | T | Intergenic | - | rs447255995 |
| 20 | 38831831 | G | A | Intergenic | - | - |
| 20 | 38832423 | C | T | Intergenic | - | rs211110735 |
| 20 | 38832840 | G | A | Intergenic | - | rs208606354 |
| 20 | 38832929 | A | C | Intergenic | - | rs210338782 |
| 20 | 38832973 | T | A | Intergenic | - | rs207695936 |
| 20 | 38832986 | C | T | Intergenic | - | rs208707838 |
| 20 | 38833043 | C | T | Intergenic | - | rs210841404 |
| 20 | 38833473 | G | A | Intergenic | - | rs208130139 |
| 20 | 38833569 | T | C | Intergenic | - | rs207783642 |
| 20 | 38833634 | T | A | Intergenic | - | rs209431885 |
| 20 | 38833687 | T | C | Intergenic | - | rs210629628 |
| 20 | 38834290 | T | C | Intergenic | - | rs382329987 |
| 20 | 38834554 | A | G | Intergenic | - | rs210856316 |
| 20 | 38834783 | C | G | Intergenic | - | rs210224871 |
| 20 | 38834941 | C | T | Intergenic | - | rs211468481 |
| 20 | 38835009 | C | T | Intergenic | - | rs208423357 |
| 20 | 38835572 | G | A | Intergenic | - | rs207692247 |
| 20 | 38835646 | G | A | Intergenic | - | rs209331338 |
| 20 | 38835657 | TG | T | Intergenic | - | rs434751839 |
| 20 | 38835662 | A | G | Intergenic | - | rs210435924 |
| 20 | 38837432 | T | TA | Intergenic | - | - |
| 20 | 38846162 | G | A | Intergenic | - | rs1115532614 |
| 20 | 38860697 | G | A | Intergenic | - | rs208124007 |
| 20 | 38861551 | A | G | Intergenic | - | - |
| 20 | 38876849 | C | T | Intergenic | - | rs207999839 |
| 20 | 38885547 | T | A | Intergenic | - | rs208430393 |
| 20 | 38889045 | C | A | Intergenic | - | rs208982333 |
| 20 | 38988043 | C | T | Intergenic | - | - |
| 20 | 39060038 | AG | A | Intronic | *PRLR* | - |
| 20 | 39790189 | A | C | Missense variant | *SLC45A2* | - |

Supplementary Table S4. Candidate variants not previously reported (no RefSeq ID) as determined from a query of the University of Missouri database of over 5,500 cattle. Given is the count of unique observations and the number of breeds in which the variant was observed. All positions are with respect to (ARS-UCD1.2; chromosome 20 (NC_037347.1)).

| Chr | Position (bp) | Reference | Alternative | No. of Animals | No. of Breeds |
| --- | --- | --- | --- | --- | --- |
| 20 | 37995958 | T | C | 175 | 18 |
| 20 | 38006184 | C | T | 1 | 1 (Hereford) |
| 20 | 38040367 | G | T | 253 | 22 |
| 20 | 38052636 | G | A | 1 | 1 (Hereford) |
| 20 | 38156214 | TC | T | 0 | 0 |
| 20 | 38541369 | A | G | 3 | 1 (Hereford) |
| 20 | 38638882 | T | G | 71 | 14 |
| 20 | 38655483 | T | C | 3 | 1 (Hereford) |
| 20 | 38804373 | CT | C | 465 | 35 |
| 20 | 38804752 | G | GA | 220 | 21 |
| 20 | 38807353 | C | T | 418 | 32 |
| 20 | 38807365 | A | G | 410 | 32 |
| 20 | 38807403 | AAG | A | 361 | 27 |
| 20 | 38831831 | G | A | 579 | 56 |
| 20 | 38837432 | T | TA | 480 | 31 |
| 20 | 38861551 | A | G | 4 | 2 |
| 20 | 38988043 | C | T | 149 | 4 |
| 20 | 39060038 | AG | A | 153 | 5 |
| 20 | 39790189 | A | C | 0 | 0 |

Supplementary Table S5. The complete genotypes of the 227 Dexter cattle studied, and their counts, considering variation in *MC1R, TYRP1,* and *SLC45A2*.

| **Phenotype** | **MC1R** | **TYRP1** | **SLC45A2** | **Count** | **Total** |
| --- | --- | --- | --- | --- | --- |
| **Black** | ED/ED | B/B | A/C | 8 | 100 |
|  | ED/ED | B/b | A/A | 42 |  |
|  | ED/E+ | B/b | A/C | 6 |  |
|  | ED/E+ | B/b | A/A | 4 |  |
|  | ED/e | B/B | A/A | 23 |  |
|  | ED/e | B/B | A/C | 3 |  |
|  | ED/ev1 | B/B | A/C | 7 |  |
|  | ED/ev1 | B/b | A/A | 7 |  |
| **Dun** | ED/ED | b/b | A/A | 24 | 55 |
|  | ED/ED | b/b | A/C | 1 |  |
|  | ED/E+ | b/b | A/A | 7 |  |
|  | ED/e | b/b | A/A | 17 |  |
|  | ED/ev1 | b/b | A/A | 4 |  |
|  | ED/ev1 | b/b | A/C | 2 |  |
| **Chocolate** | ED/ED | B/B | C/C | 1 | 8 |
|  | ED/E+ | B/B | C/C | 5 |  |
|  | ED/e | B/B | C/C | 1 |  |
|  | ED/ev1 | B/b | C/C | 1 |  |
| **Red** | E+/E+ | B/B | A/A | 5 | 58 |
|  | E+/E+ | B/B | A/C | 7 |  |
|  | E+/e | B/B | A/C | 6 |  |
|  | E+/e | b/b | A/A | 15 |  |
|  | E+/ev1 | B/B | A/A | 1 |  |
|  | e/e | B/b | A/A | 15 |  |
|  | e/e | B/B | A/C | 1 |  |
|  | e/ev1 | B/B | A/A | 2 |  |
|  | ev1/ev1 | B/B | A/C | 4 |  |
|  | ev1/ev1 | B/b | A/A | 2 |  |
| **Cream** | E+/E+ | B/B | C/C | 2 | 6 |
|  | E+/e | B/B | C/C | 3 |  |
|  | e/e | B/B | C/C | 1 |  |

Supplementary Figure F1. A portion of the SLC45A2 protein highlighting the amino acid conservation across species. The position impacted due to the Dexter mutation (XP_002696432.2: p:Gln133Pro) is outlined in red.
